# Serum Interleukin-18 Levels Are Associated with Physical Activity in Japanese Men

**DOI:** 10.1371/journal.pone.0081497

**Published:** 2013-12-11

**Authors:** Kanae Oda, Nobuyuki Miyatake, Noriko Sakano, Takeshi Saito, Motohiko Miyachi, Izumi Tabata, Takeyuki Numata

**Affiliations:** 1 Department of Hygiene, Faculty of Medicine, Kagawa University, Kita, Kagawa, Japan; 2 Health Promotion and Exercise Program, National Institute of Health and Nutrition, Shinjuku, Tokyo, Japan; 3 Faculty of Sport and Health Science, Ritsumeikan University, Kusatsu, Shiga, Japan; 4 Okayama Southern Institute of Health, Okayama Health Foundation, Kita, Okayama, Japan; Universidad Pablo de Olavide, Centro Andaluz de Biología del Desarrollo-CSIC, Spain

## Abstract

**Objective:**

To investigate the link between serum interleukin-18 (IL-18) levels and physical activity in Japanese men.

**Methods:**

A total of 81 men (45.7±17.6 years old) was enrolled in this cross-sectional investigation study. We assessed anthropometric and body composition parameters. Serum IL-18 levels, physical activity by uniaxial accelerometers, peak oxygen uptake and metabolic risk parameters were also evaluated.

**Results:**

Serum IL-18 levels were 179.4±84.7 pg/mL. Physical activity evaluated by Σ[metabolic equivalents × h per week (METs⋅h/w)]was significantly and negatively correlated with serum IL-18 levels (r = −0.252, *p* = 0.0235). These associations remained even after adjusting for age, peak oxygen uptake and other confounding factors.

**Conclusion:**

Serum IL-18 levels were closely associated with physical activity independent of peak oxygen uptake in Japanese men.

## Introduction

Interleukin-18 (IL-18) is a proinflammatory cytokine secreted from mononuclear cells [1]–[3]. Although high serum IL-18 levels were reported in patients with rheumatoid arthritis [4] and adult-onset Still’s disease [5], they were strong predictor of death in patients with coronary artery disease [6] and acute ischemic stroke [7]. In addition, serum IL-18 levels were elevated in patients with diabetes mellitus [8] and diabetic nephropathy [9], which were in the state of low grade inflammation (microinflammation). Thus, serum IL-18 levels might be important factor and predictor in the process of atherosclerosis.

Regular physical activity increases high density lipoprotein (HDL) cholesterol and reduces resting blood pressure, fasting blood glucose, triglycerides, abdominal fat and insulin responses to an oral glucose challenge test [10]–[13]. Sawada *et al* [14] reported that low cardiorespiratory fitness was linked to cancer mortality in Japanese men. Sandvik *et al* [15] also showed that physical fitness was a graded, independent, long-term predictor of mortality from cardiovascular causes in healthy, middle-aged men. Maximal oxygen uptake is generally considered an accurate and reliable parameter. In the Exercise and Physical Activity Reference for Health Promotion 2006, established by the Ministry of Health Labour and Welfare of Japan in 2006, maximal oxygen uptake was considered to be the most significant element of physical fitness related to health promotion, and the recommended reference value for maximal oxygen uptake to prevent lifestyle-related disease was reported [16]. Taken together, physical activity and/or physical fitness may reduce serum IL-18 levels resulting in protective effect on atherosclerosis.

However, the link between serum IL-18 levels and physical activity, maximal oxygen uptake still remains unknown. Therefore, in this study, we evaluated the relationship between serum IL-18 levels and physical activity and/or physical fitness in Japanese men.

## Methods

### 2.1. Subjects

We enrolled 81 men (45.7±17.6 years old) who met the following criteria: (1)wanted to volunteer in this cross-sectional investigation study at Okayama Southern Institute of Health, Okayama Health Foundation, Okayama, Japan; (2)had received anthropometric, physical activity, peak oxygen uptake, blood pressure (BP) measurements and blood examinations including serum IL-18 levels; (3)received no medications for diabetes, hypertension, and/or dyslipidemia; and (4)provided written informed consent (**Table 1**).

**Table 1:** Clinical characteristics of enrolled subjects.

	Mean±SD	Minimum	Maximum
Number of subjects	81		
Age (year)	45.7±17.6	21	69
Height (cm)	170.6±6.0	160.3	187.3
Body weight (kg)	66.2±9.6	48.3	96.4
Body mass index (kg/m^2^)	22.7±3.0	17.2	34.7
Abdominal circumference (cm)	80.8±8.0	63.9	101.8
Body fat percentage (%)	19.3±5.9	8.9	34.7
Peak oxygen uptake (mL/kg/min)	37.1±9.4	17.2	61.9
Peak work rate (watt)	197.4±53.9	92	354
Physical activity (METs⋅h/w)	12.7±8.4	0.8	43.2
Systolic blood pressure (mmHg)	133.1±15.3	103.0	180.0
Diastolic blood pressure (mmHg)	82.9±11.7	56.0	111.0
Blood profile			
IL-18 (pg/mL)	179.4±84.7	22.9	527.9
Triglyceride (mg/dL)	98.8±57.0	33.0	434.0
HDL cholesterol (mg/dL)	55.7±14.2	32.0	118.0
Blood glucose (mg/dL)	92.6±9.3	61.0	126.0
Number of subjects withsmoking habits (%)	38 (46.9%)		

IL-18: Interleukin-18.

⋅h/w: Σ[metabolic equivalents × h per week (METs⋅h/w)]. METs

Ethical approval for the study was obtained from the Ethical Committee of Okayama Health Foundation, Okayama, Japan.

### 2.2. Blood Sampling and Assays

After the subjects fasted overnight for 10–12 hours, blood samples were collected in order to determine the serum levels of IL-18, high density lipoprotein (HDL) cholesterol, triglycerides (L Type Wako Triglyceride⋅H, Wako Chemical, Osaka, Japan), and blood glucose. Serum IL-18 levels were measured using a commercially available enzyme-linked immunosorbent assay (MBL, Nagoya, Japan). Blood glucose was measured by the glucose-oxidant method.

### 2.3 Anthropometric and Body Composition Measurements

Anthropometric and body compositions were evaluated based on the following parameters: height, body weight, abdominal circumference and body composition. The abdominal circumference was measured at the umbilicus in standing subjects after a normal exhalation [17]. Body mass index (BMI) was calculated by weight/[height]^2^ (kg/m^2^). The body fat percentage was measured by DEXA (QDR4500, Hologic Inc., Waltham, MA, USA), which is accepted as an accurate standard [18]. The DEXA measurement consisted of a whole body scan using an array beam [19]. The subjects removed all metal objects, and were positioned in the supine position with their hands placed on either side of the body and their legs held 10 cm apart according to the specifications of the manufacturer. All scans were analyzed according to the manufacturer’s instructions [20].

### 2.4. Physical Activity

Physical activity was measured by the Kenz Lifecorder (LC; SUZUKEN Co Ltd, Nagoya, Japan) which is a recent addition to the growing number of uniaxial accelerometer options; it offers comparable instrument outputs with several potentially attractive features for researchers and practitioners. The LC displays reasonable estimates of physical activity intensity and energy expenditures under controlled conditions on a treadmill [21], over 24 h of typical daily activities undertaken in a respiratory chamber [21], and in a free-living environment using double-labeled water as the criterion method [22]. Furthermore, when compared with many other accelerometers, the LC can potentially simplify the data interpretation process by reducing the time spent and the need for advanced technical expertise or software programs [23]. The subjects were taught how to use the instrument, and were told to wear it on their belt or waist band at the right midline of the thigh from the moment they got up until they went to bed except while bathing or swimming, for seven consecutive days [24]. The activity monitor was firmly attached to their clothes at the waist by a clip.

### 2.5. Exercise Testing

Peak oxygen uptake was measured using a maximal graded exercise test with bicycle ergometers (Excalibur V2.0, Lode BV, Groningen, Netherlands). The initial work load was 30–60 w, and the work rate was increased thereafter by 15 w/min until the subject could not maintain the required pedaling frequency (60 rpm) [25]. During the latter stages of the test, each subject was verbally encouraged by the test operators to give their maximal effort. In addition, an ECG was monitored continuously while recording the heart rate (HR). The expired gas was collected, and the rates of oxygen consumption (VO_2_) and carbon dioxide production (VCO_2_) were measured breath-by-breath using a cardiopulmonary gas exchange system (Oxycon Alpha, Mijnhrdt B.V., Netherlands). The achievement of peak oxygen uptake was accepted if the following two conditions were met: the subject’s maximal HR was >95% of the age-predicted maximal HR (220 – age), and the VO_2_ curve showed a leveling off. In addition, the observed maximal work rate during the testing was used for this analysis.

### 2.6. Blood Pressure (BP) Measurements at Rest

Resting systolic and diastolic BP (SBP and DBP) were measured indirectly using a mercury sphygmomanometer placed on the right arm of the seated participant after at least 15 minutes of rest.

### 2.7. Cigarette Smoking

The data on cigarette smoking was obtained through structured interviews conducted by public health nurses trained for this study. The subjects were asked if they currently smoked cigarettes. When the answer was “yes”, they were classified as current smokers. In the case of a “no” answer, they were classified as non smokers. We could not classify those who used to smoke but had since stopped smoking.

### 2.8. Statistical Analysis

All data are expressed as means ± SD values. Pearson’s correlation coefficients were calculated and used to test the significance of the linear relationship between continuous parameters: where *p*<0.05 was considered statistically significant. A multiple logistic and multiple stepwise regression analysis were also performed to test the relationship between serum IL-18 levels and physical activity, and between serum IL-18 levels and peak oxygen uptake.

## Results

The measurements of parameters were summarized in Table 1. Serum IL-18 levels in enrolled men was 179.4±84.7 pg/mL. Physical activity evaluated by the LC was 12.7±8.4 METs⋅h/w (**Table 1**).

**Figure 1:**
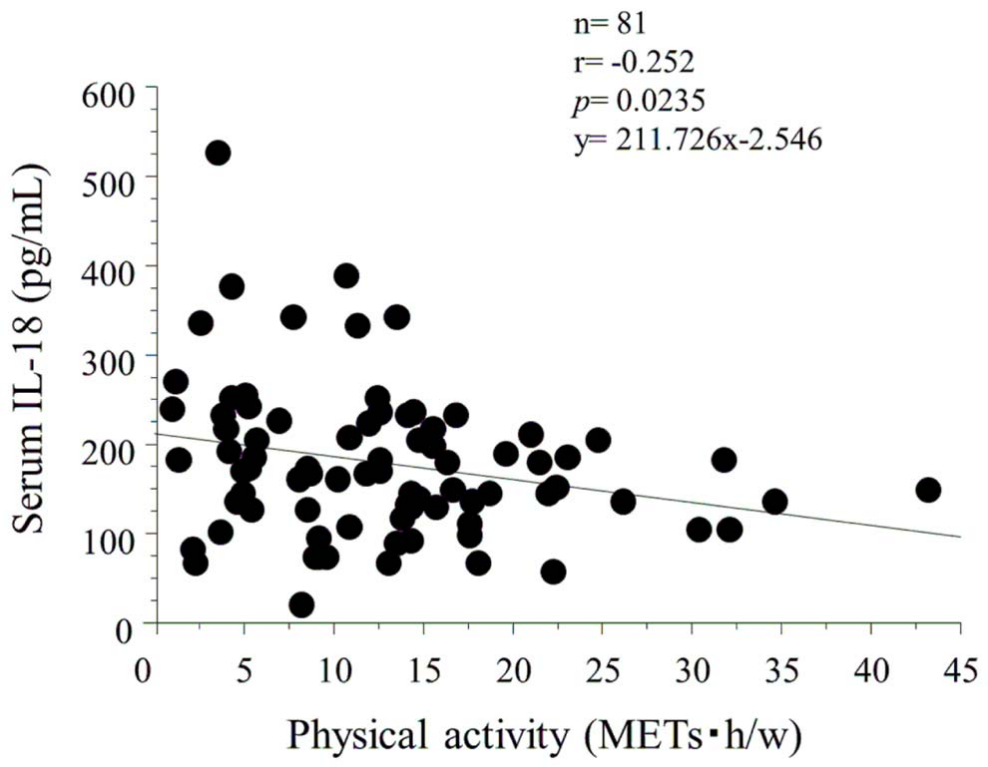
Simple correlation analysis between serum IL-18 levels and physical activity by using uniaxial accelerometer. Serum IL-18 levels were significantly and negatively correlated with physical activity (r = −0.252, *p* = 0.0235).

The simple correlation analysis between serum IL-18 levels and clinical parameters was evaluated (**Table 2**). Serum IL-18 levels were significantly and negatively correlated with physical activity (r = −0.252, *p* = 0.0235) (**Table 2** **,** **Fig. 1**). However, significant relationships between serum IL-18 levels and other parameters were not noted.

**Table 2:** Simple correlation analysis between serum IL-18 levels and clinical parameters.

	r	*p*
Age (year)	0.145	0.1978
Height (cm)	−0.181	0.1055
Body weight (kg)	−0.061	0.5893
Body mass index (kg/m^2^)	0.016	0.8870
Abdmonal circumference (cm)	0.108	0.3369
Body fat percentage (%)	0.099	0.3812
Peak oxygen uptake (mL/kg/min)	−0.189	0.0906
Peak work rate (watt)	−0.184	0.0997
Physical activity (METs⋅h/w)	−0.252	**0.0235**
Systolic blood pressure (mmHg)	0.199	0.0752
Diastolic blood pressure (mmHg)	0.215	0.0543
Blood profile		
Triglyceride (mg/dL)	0.156	0.1655
HDL cholesterol (mg/dL)	−0.117	0.2993
Blood glucose (mg/dL)	0.133	0.2380

IL-18: Interleukin-18.

⋅h/w: Σ[metabolic equivalents × h per week (METs⋅h/w)]. METs

Table 3 showed the results by multiple logistic regression analysis about serum IL-18 levels, according to quartiles of physical activity. The odds ratio of serum IL-18 levels according to quartiles of physical activity was 0.214 (not adjusted), and 0.215 even after adjusting for age, peak oxygen uptake, BMI, abdominal circumference and cigarette smoking habit. However, the relation was attenuated after adjusting for age, peak oxygen uptake, BMI, abdominal circumference, cigarette smoking habit and body fat percentage (**Table 3**). In turn, the odds ratio of serum IL-18 levels according to quartiles of peak oxygen uptake was not at a significant level (data not shown).

**Table 3:** Odds ratio of serum IL-18 levels according to quartiles of physical activity.

	Quartiles of physicalactivity (METs⋅h/w)			
	Q1	Q2	Q3	Q4
Number of subjects	20	20	20	21
Mean ± SD	3.6±1.4	9.0±1.8	14.0±1.1	23.6±7.1
Model 1	1	0.351(0.096–1.287)	0.524(0.143–1.923)	**0.214(0.057–0.801)**
Model 2	1	0.355(0.096–1.306)	0.502(0.134–1.887)	**0.203(0.052–0.788)**
Model 3	1	0.368(0.099–1.364)	0.529(0.140–2.002)	**0.230(0.058–0.918)**
Model 4	1	0.337(0.086–1.318)	0.604(0.152–2.394)	**0.215(0.049–0.942)**
Model 5	1	0.395(0.093–1.674)	0.669(0.163–2.749)	0.255(0.053–1.218)

⋅h/w: Σ[metabolic equivalents × h per week (METs⋅h/w)]. METs

Data were analyzed by multiple logistic regression analysis.

Model 1: Not adjusted.

Model 2: Adjusted for age.

Model 3: Adjusted for age and peak oxygen uptake.

Model 4: Adjusted for age, peak oxygen uptake, BMI, abdominal circumference and cigarette smoking habit.

Model 5: Adjusted for age, peak oxygen uptake, BMI, abdominal circumference, cigarette smoking habit and body fat percentage.

Finally, we used stepwise multiple regression analysis to evaluate the effect of clinical parameters *i.e.* age, BMI, abdominal circumference, body fat percentage, physical activity and peak oxygen uptake on serum IL-18 levels, and found that age and physical activity were significant (Serum IL-18 levels = 168.141+1.150 (age) –3.255 (physical activity), r^2^ = 0.093, *p* = 0.0084).

## Discussion

In this study, we accurately evaluated the relationship between serum IL-18 levels and physical activity using uniaxial accelerometer in Japanese men for the first time. Physical activity was closely associated with serum IL-18 levels, even after adjusting for confounding factors such as peak oxygen uptake.

A major mechanism of cardiovascular events mediated by IL-18 is decreased stability of plaque. Carotid intima-media thickness (IMT) measured by carotid ultrasound is a useful tool for assessing cardiovascular diseases in diabetes mellitus [26], and carotid IMT in patients with high IL-18 showed a greater thickness than in patients with normal IL-18 [27]. According to the relationship between IL-18 and exercise, Leick *et al* reported that exercise training reduced adipose tissue IL-18 mRNA content by 20% in both sexes after 8 weeks of exercise training in obese subjects [28]. Stensvold D *et al* also showed that serum IL-18 levels were reduced by 43% after aerobic interval training in 11 inactive men and women with metabolic syndrome [29]. Exercise training reduced IL-18 with a 6-month aerobic exercise training program (four times/week, 40–60 min/session) [30]. In this study, although our study was cross-sectional study, significant relationships between serum IL-18 levels and physical activity by using uniaxial accelerometer were noted. The relationships still remained even after adjusting for confounding factors (except for body fat percentage) by logistic regression analysis. In addition, by stepwise multiple regression analysis, only age and physical activity were significant determinant factors of serum IL-18 levels. It is noteworthy that the serum IL-18 levels were closely linked to physical activity independent of physical fitness. Brun JM *et* *al* reported that plasma IL-18 was associated with changes in insulin resistance but not with BMI [31]. Daily walking rather than increasing physical fitness was closely associated with improving insulin resistance [13]. Taken together, increasing physical activity, independent of physical fitness, may reduce serum IL-18 levels in some healthy Japanese men.

Potential limitations still remain in this study. First, our study was a cross sectional but not a longitudinal study. Second, 81 men in our study voluntarily underwent measurements: they were therefore more likely to be health-conscious as compared with the average person. Third, we could not evaluate in women and also could not show a clear mechanism between serum IL-18 levels and physical activity. However, it seems reasonable to suggest that promoting physical activity might result in reducing serum IL-18 levels in some healthy Japanese men. To show this, further prospective and larger sample size studies are urgently required in the Japanese population.
